# Second-generation cryoballoon versus contact force radiofrequency ablation for atrial fibrillation: an updated meta-analysis of evidence from randomized controlled trials

**DOI:** 10.1038/s41598-021-96820-8

**Published:** 2021-09-09

**Authors:** Chenxia Wu, Xinyi Li, Zhengtian Lv, Qian Chen, Yang Lou, Wei Mao, Xinbin Zhou

**Affiliations:** 1 Department of Cardiology, First Affiliated Hospital of Zhejiang Chinese Medical University, Hangzhou, 310006 China; 2 The First College of Clinical Medicine, Zhejiang Chinese Medical University, Hangzhou, China; 3 Department of Cardiology, Qingdao Hospital of Traditional Chinese Medicine (Qingdao Hiser Hospital), Qingdao, 266000 China

**Keywords:** Cardiac device therapy, Interventional cardiology

## Abstract

Catheter ablation has been recommended for patients with symptomatic atrial fibrillation (AF), with pulmonary vein isolation being the cornerstone of the ablation procedure. Newly developed technologies, such as cryoballoon ablation with a second-generation cryoballoon (CB2) and the contact force radiofrequency (CF-RF) ablation, have been introduced in recent years to overcome the shortcomings of the widely used RF ablation approach. However, high-quality results comparing CB2 and CF-RF remain controversial. Thus, we conducted this meta-analysis to assess the efficacy and safety between CB2 and CF-RF using evidence from randomized controlled trials (RCTs). Databases including Embase, PubMed, the Cochrane Library, and ClinicalTrials.gov were systematically searched from their date of inception to January 2021. Only RCTs that met the inclusion criteria were included for analysis. The primary outcome of interest was freedom from atrial tachyarrhythmia (AT) during follow-up. Secondary outcomes included procedure-related complications, procedure time and fluoroscopy time. Six RCTs with a total of 987 patients were finally enrolled. No significant differences were found between CB2 and CF-RF in terms of freedom from AT (relative risk [RR] = 1.03, 95% confidence interval [CI] 0.92–1.14, *p* = 0.616) or total procedural-related complications (RR = 1.25, 95% CI 0.69–2.27, *p* = 0.457). CB2 treatment was associated with a significantly higher risk of phrenic nerve palsy (PNP) than CF-RF (RR = 4.93, 95% CI 1.12–21.73, *p* = 0.035). The occurrences of pericardial effusion/tamponade and vascular complications were comparable between the CB2 and CF-RF treatments (RR = 0.41, *p* = 0.398; RR = 0.82, *p* = 0.632). In addition, CB2 treatment had a significantly shorter procedure time than CF-RF (weighted mean difference [WMD] = − 20.75 min, 95% CI − 25.44 ~ − 16.05 min, *P* < 0.001), whereas no difference was found in terms of fluoroscopy time (WMD = 4.63 min, *p* = 0.179). CB2 and CF-RF treatment are comparable for AF patients regarding freedom from AT and procedure-related complications. Compared to CF-RF, CB2 treatment was associated with a shorter procedure time but a higher incidence of PNP. Further large-scale studies are warranted to compare these two techniques and provide an up-to-date recommendation.

## Introduction

Atrial fibrillation (AF) is the most common cardiac arrhythmia and is associated with an increased risk of stroke, heart failure and death^1^. Antiarrhythmic drugs (AADs) therapy, including rhythm control and rate control strategies, has been the primary treatment for AF for decades. Nevertheless, the effectiveness of AADs is limited, and the adverse effects may negate the benefits of the sinus rhythm. Thus, other strategies to maintain sinus rhythm have been developed, such as catheter ablation^2,3^.

Although the recent CABANA trial has shown that, the strategy of AF catheter ablation was not superior compared with medical therapy in reducing the primary composite outcome of death, disabling stroke, serious bleeding, or cardiac arrest^3^, multiple studies have demonstrated that AF catheter ablation is superior to AADs for maintaining sinus rhythm and improving arrhythmia-related symptoms, with comparable complication rates^4,5^.

In addition, for AF patients with heart failure (HF) and reduced left ventricular ejection fraction (LVEF), several studies, including a subgroup analysis of the CABANA trial, have shown a more remarkable improvement in LVEF, and a reduction in all-cause mortality and hospitalizations with catheter ablation treatment^3,6,7^.

For symptomatic AF patients, catheter ablation has been recommended by guidelines to restore and maintain sinus rhythm, and pulmonary vein isolation (PVI) has been the cornerstone of ablation procedures^2^. Although point-by-point radiofrequency (RF) ablation has been widely used, it remains a complex and time-consuming procedure. To overcome these limitations, the newly updated and developed technologies, such as cryoballoon ablation (CBA) with a second-generation cryoballoon (CB2) and contact force radiofrequency (CF-RF) ablation, have been introduced in recent years.

Although many studies have been performed to compare CB2 and CF-RF, high-quality evidence is lacking, and the results remain controversial^8–11^. Thus, we performed this meta-analysis to assess the efficacy and safety of CB2 and CF-RF based on evidence from randomized controlled trials (RCTs).

## Materials and methods

### Search strategy and selection criteria

We systematically searched databases including Embase, PubMed, the Cochrane Library, and ClinicalTrials.gov from their date of inception to January 2021. The following keywords and variants thereof were used: “atrial fibrillation”, “cryoballoon”, “cryoablation”, and “radiofrequency”. Additionally, the reference lists of the included articles and relevant reviews were searched for potentially relevant studies.

Studies were required to meet the following criteria to be included: (1) RCTs, (2) full-text articles in English, (3) comparisons between CB2 and CF-RF for AF patients, (4) outcomes of interest were reported, and (5) the length of follow-up was at least three months.

### Data collection and quality assessment

Two investigators performed the data extraction and quality assessment independently, and discrepancies were resolved by consensus. The following data were extracted: publication information, participant characteristics, AF type, follow-up duration, and outcomes of interest. The Cochrane Collaboration tool was applied to evaluate the quality of the included studies^12^.

### Outcomes

The primary outcome of interest was freedom from atrial tachyarrhythmia (AT), including AF, atrial flutter or atrial tachycardia during follow-up. Secondary outcomes included procedure time, fluoroscopy time and procedure-related complications, including death, pericardial effusion, tamponade, pericarditis, HF exacerbation, stroke, arteriovenous fistula, haematoma requiring intervention, pseudoaneurysm requiring intervention, phrenic nerve palsy (PNP), and esophageal perforation or injury.

### Statistical analysis

STATA software (STATA Corporation, TX, USA, version 12.0) was applied for statistical analyses. Relative risk (RR) or weighted mean difference (WMD) and the 95% confidence interval (CI) were calculated to demonstrate the overall result. Heterogeneity was estimated by Cochran’s Q test, and I^2^ > 50% was considered statistically significant. A random-effects model was applied and if there was significant heterogeneity, the possible reasons were investigated. Additional subgroup analysis was also performed for paroxysmal and persistent AF. Publication bias for the primary outcome was assessed graphically using funnel plots and statistically using Egger’s and Begg’s tests. A two-sided *p* value < 0.05 was considered statistically significant.

## Results

### Eligible studies and characteristics

**Figure 1:**
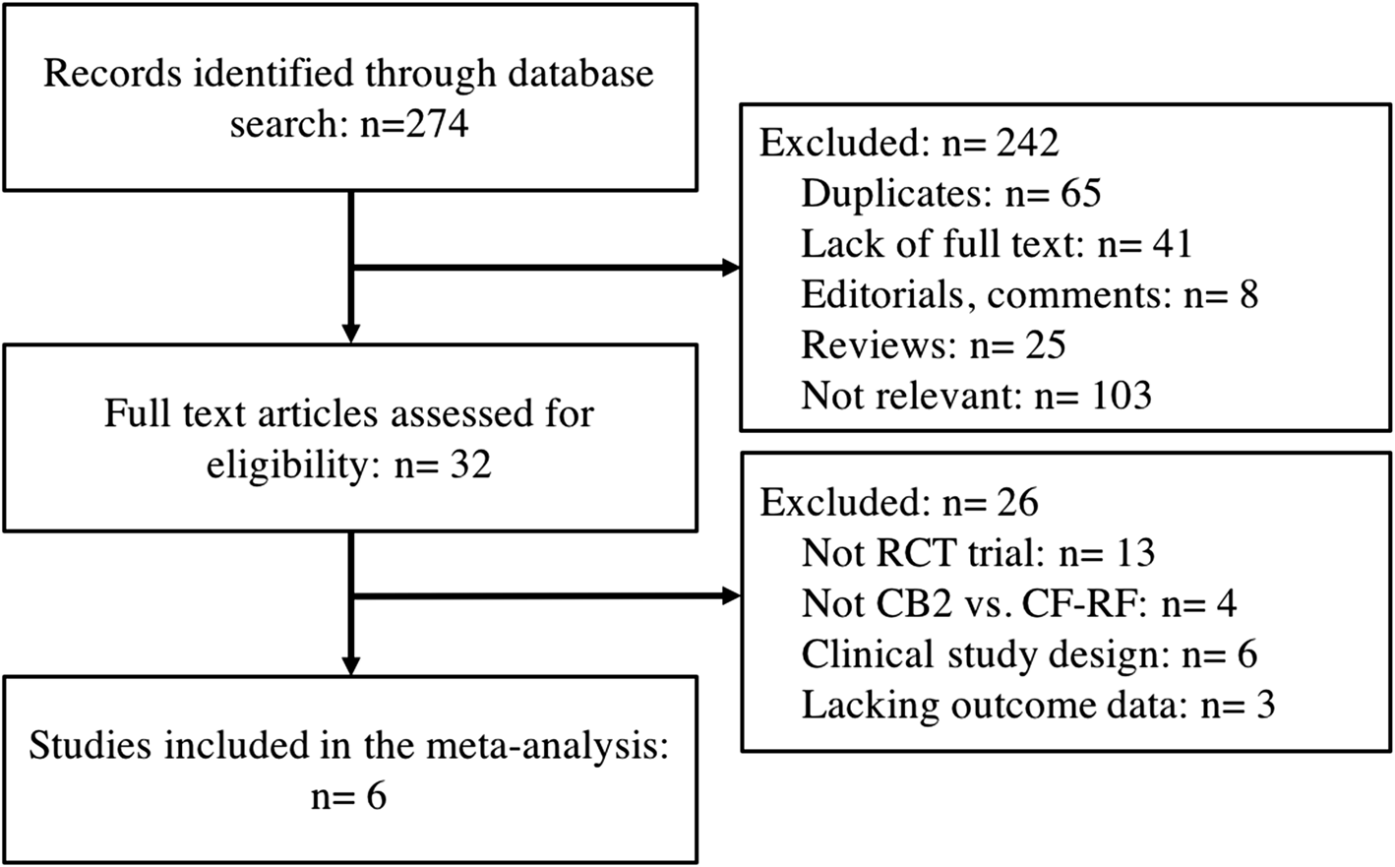
Flow chart of the systematic literature research.

A total of 274 potentially relevant studies were identified in the initial search, of which 32 studies were further assessed. Finally, six RCTs with a total of 987 patients were included in the meta-analysis^8,9,13–16^ (Fig. 1). No additional studies were identified. The baseline characteristics of the included studies are presented in Tables 1 and 2. Briefly, across the trials, 574 patients were classified in the CB2 group and 413 in the CF-RF group. The mean age of the patients ranged from 58.6 to 65 years. Two studies^8,9^ included both paroxysmal AF (PAF) and persistent AF (PerAF) patients, and the rest included only PAF patients^13–16^ (Table 1).

**Table 1:** Baseline characteristics of the included trials.

Study	Year	Patients (n)		Mean age (years)	Male,%	Mean Lad (mm)	Mean LVEF (%)	Hypertension (%)	DM(%)	CAD(%)	Follow-up (months)
		PAF n (%)	PerAF n (%)								
Andrade	2019	327 (94.5)	19 (5.5)	59	67	37.8	59.2	34.7	NR	7.2	12
You	2019	140 (100)	0 (0)	58.6	56.4	NR	NR	56.5	17.9	NR	12
Giannopoulos	2019	120 (100)	0 (0)	60	NR	40.5	60	49.2	12.5	6.7	6
Buist	2018	229 (85.1)	40 (14.9)	58.9	71	NR	NR	40.8	23	NR	12
Watanabe	2018	52 (100)	0 (0)	65	72	40.5	60.5	60	16	NR	12
Gunawardene	2018	60 (100)	0 (0)	59.7	70	NR	59.5	55	NR	NR	12

PAF = paroxysmal atrial fibrillation, PerAF = persistent atrial fibrillation, LAd = left atrial diameter, LVEF = left ventricular ejection fraction, DM = diabetes mellitus, CAD = coronary artery disease, NR = not reported.

**Table 2:** Procedural and monitoring protocols of the included studies.

Study	Arrhythmia monitoring methodology	Protocol of AADs after ablation	Cryoablation protocol	Radiofrequency ablation protocol
Andrade	Implantable Loop Recorder	Discontinued after the blanking period	Cryoablation was performed with a lesion duration of 4 min or 2 min	The CF targeted prior to lesion delivery was 20 g (10–40 g), with a minimum individual FTI > 400 gs
You	NR	NR	Cryoablation was performed up to 180 s and two times for each PV	Standardized RFCA procedure
Giannopoulos	NR	Discontinued after the blanking period	Cryoablation energy was applied for 240 s; if no PV signals were recorded in a vein the time of energy delivery was reduced to 180 s	Standardized RFCA procedure
Buist	ECG; 24–72 h Holter ECG	Discontinued after the blanking period	Cryo-energy applications were performed up to 240 s, with an additional number of cryo applications at the operator’s discretion	Power setting was adjusted between 30–40 w, RF application duration of 20 to 40 s, FTI > 400 gs
Watanabe	ECG; Holter ECG	Discontinued after the blanking period	A 180 s-freeze was delivered to each PV	RF energy was delivered with a maximum power of 30 W, CF of 10–15 g
Gunawardene	ECG ; 5-day Holter ECG	Discontinued after the ablation	Target application time was 240 s (332.0 + 159.3 s per PV and 1.54 + 0.76 cryo freezes per PV, as results)	RF energy was delivered with a maximum power of 30 W for 30–60 s, temperature limit of 45℃, minimal CF of 10 g

AADs = antiarrhythmic drugs, ECG = electrocardiograph, PV = pulmonary vein, CF = contact force, FTI = force–time integral, RFCA = radiofrequency catheter ablation, NR = not reported.

All the included studies had good qualities according to the Cochrane Collaboration tool. No significant publication bias was found by funnel plot or Egger’s and Begg’s tests based on the primary outcome (Egger’s: *p* = 0.551; Begg’s: *p* = 0.452).

### Primary end points

**Figure 2:**
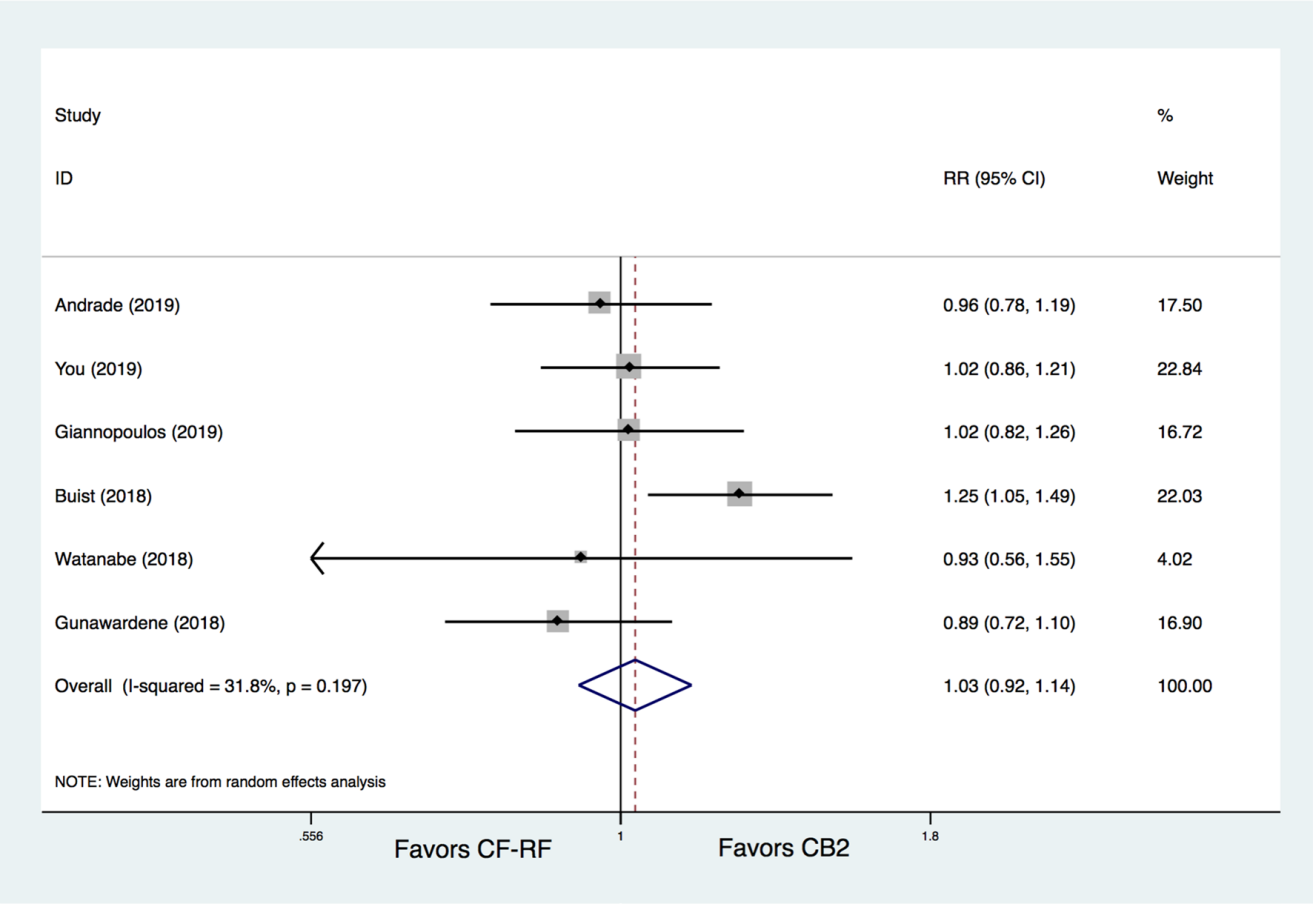
Meta-analysis for the outcome of freedom from AT. CF-RF = contact force radiofrequency ablation; CB2 = cryoballoon ablation (CBA) with second-generation cryoballoon; RR = relative risk.

**Figure 3:**
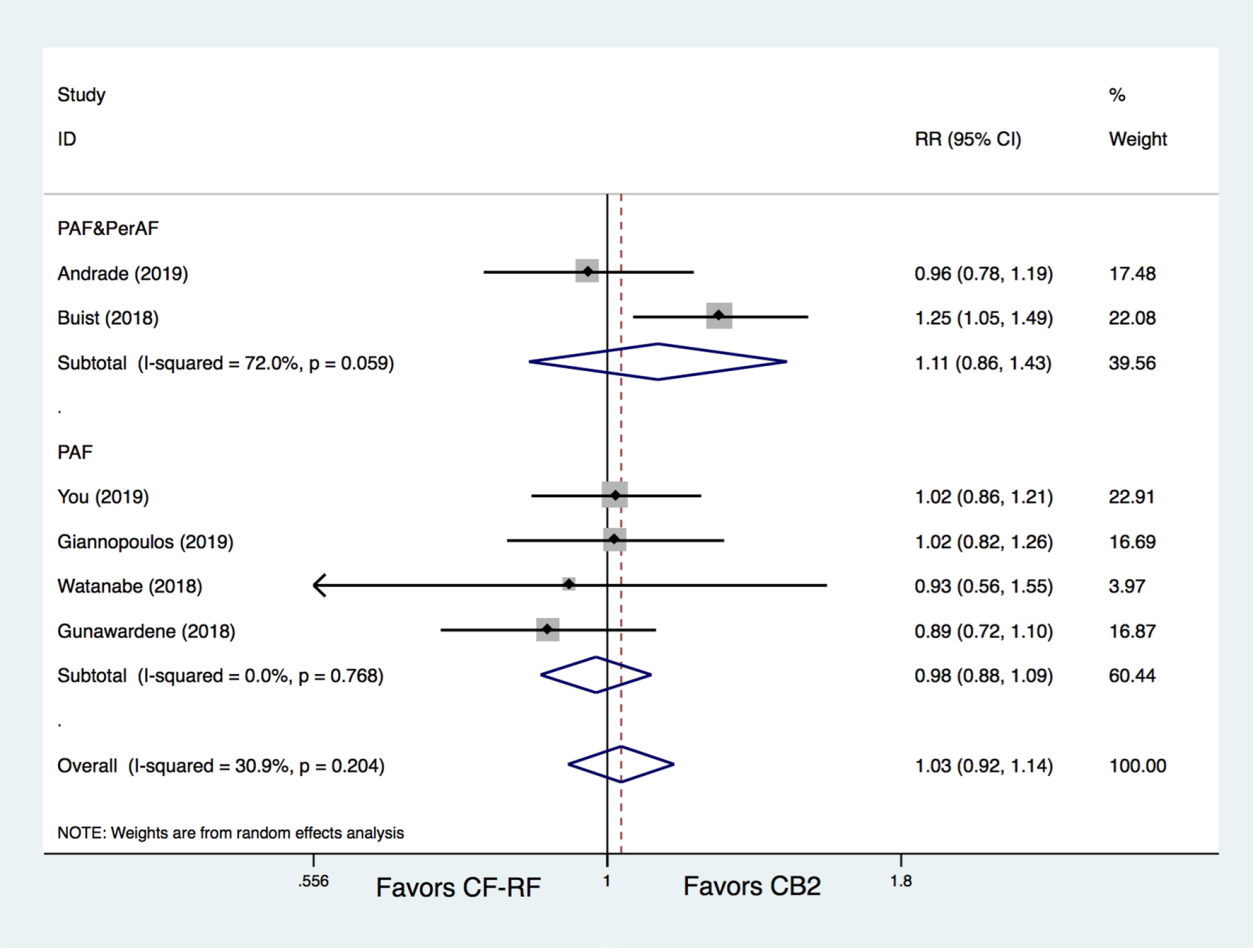
Subgroup meta-analysis for the outcome of freedom from AT. CF-RF = contact force radiofrequency ablation; CB2 = cryoballoon ablation (CBA) with a second-generation cryoballoon; PAF = paroxysmal atrial fibrillation; PerAF = persistent atrial fibrillation; RR = relative risk.

There was no significant difference between CB2 and CF-RF regarding freedom from AT (65.4% vs. 64.4%, RR = 1.03, 95% CI 0.92–1.14, *p* = 0.616). No significant heterogeneity was detected (I^2^ = 31.8%) (Fig. 2). In addition, three included trials have provided data on acute PVI rates, and there was also no significant difference between CB2 and CF-RF (99.5% vs. 99.6%, RR = 1.14, 95% CI 0.29–4.48, *p* = 0.847). Further subgroup analysis based on AF type demonstrated similar results. For the PAF only patients group, the proportion of freedom from AT was 75.1% in the CB2 group versus 76.4% in the CF-RF group (RR = 0.98, 95% CI 0.88–1.09, *p* = 0.688), and for the mixed patients group including both PAF and PerAF, it was 59.9% versus 56.5% (RR = 1.11, 95%CIs 0.86–1.43, *p* = 0.442) (Fig. 3). Additional leave-one-out analysis was performed and showed similar results, with RRs ranging from 0.975 to 1.061 (*p* > 0.05 for all the analyses). A meta-regression analysis was also conducted, and the results demonstrated that, all of the patient characteristics, such as participant number, percentages of PAF, mean age, percentages of males, mean LAd, mean LVEF, and follow-up lengths, were not significantly associated with the primary and main secondary outcomes (*p* > 0.05 for all).

### Secondary end points

**Figure 4:**
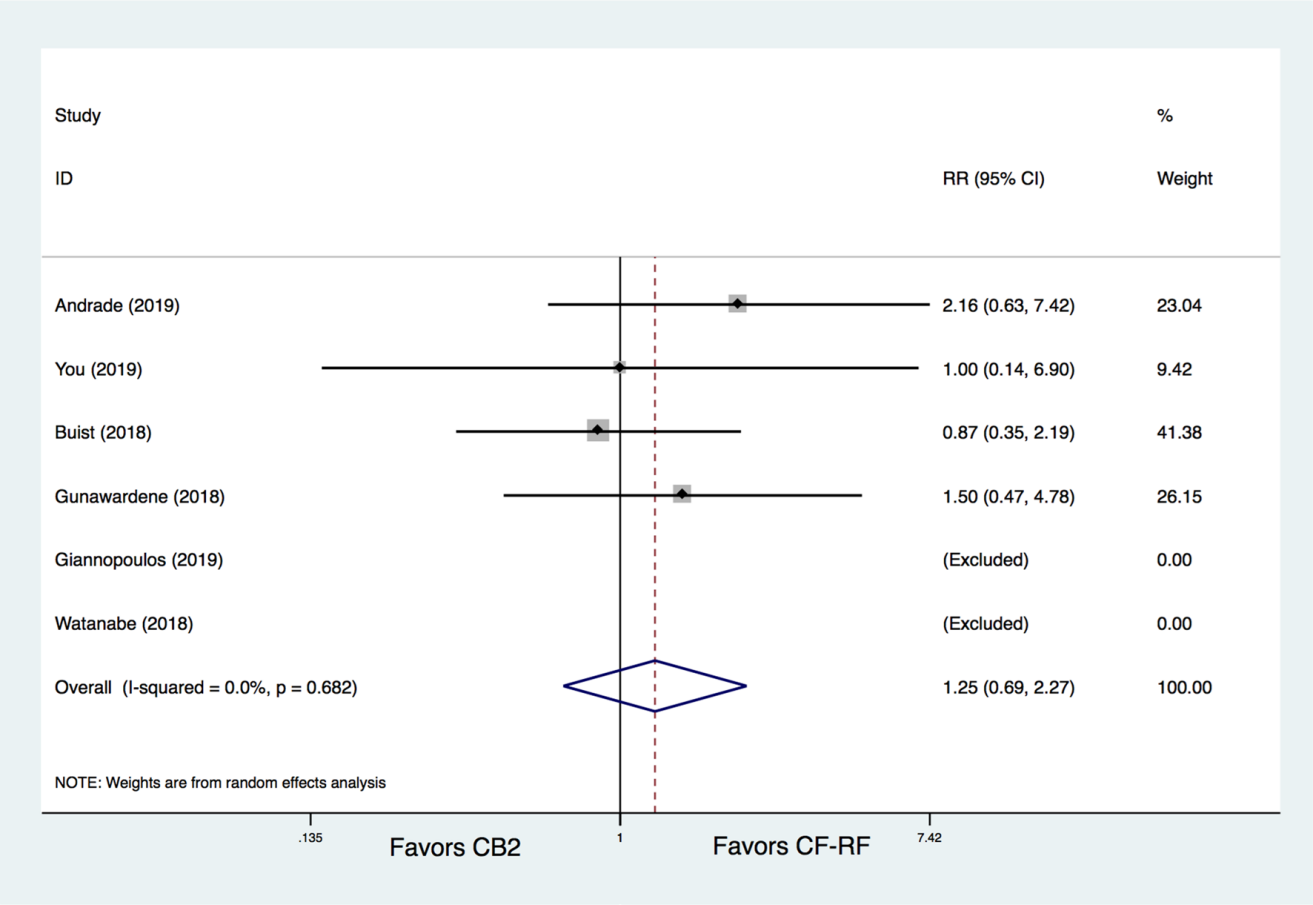
Meta-analysis for the outcome of total complications. CF-RF = contact force radiofrequency ablation; CB2 = cryoballoon ablation (CBA) with a second-generation cryoballoon; RR = relative risk.

The total procedure-related complication rates were similar between the CB2 group and CF-RF group (5.1% versus 4.4%, RR = 1.25, 95% CI 0.69–2.27, *p* = 0.457) (Fig. 4). Additional leave-one-out analysis was performed and showed similar results, with RRs ranging from 1.064 to 1.621 (*p* > 0.05 for all the analyses).

**Figure 5:**
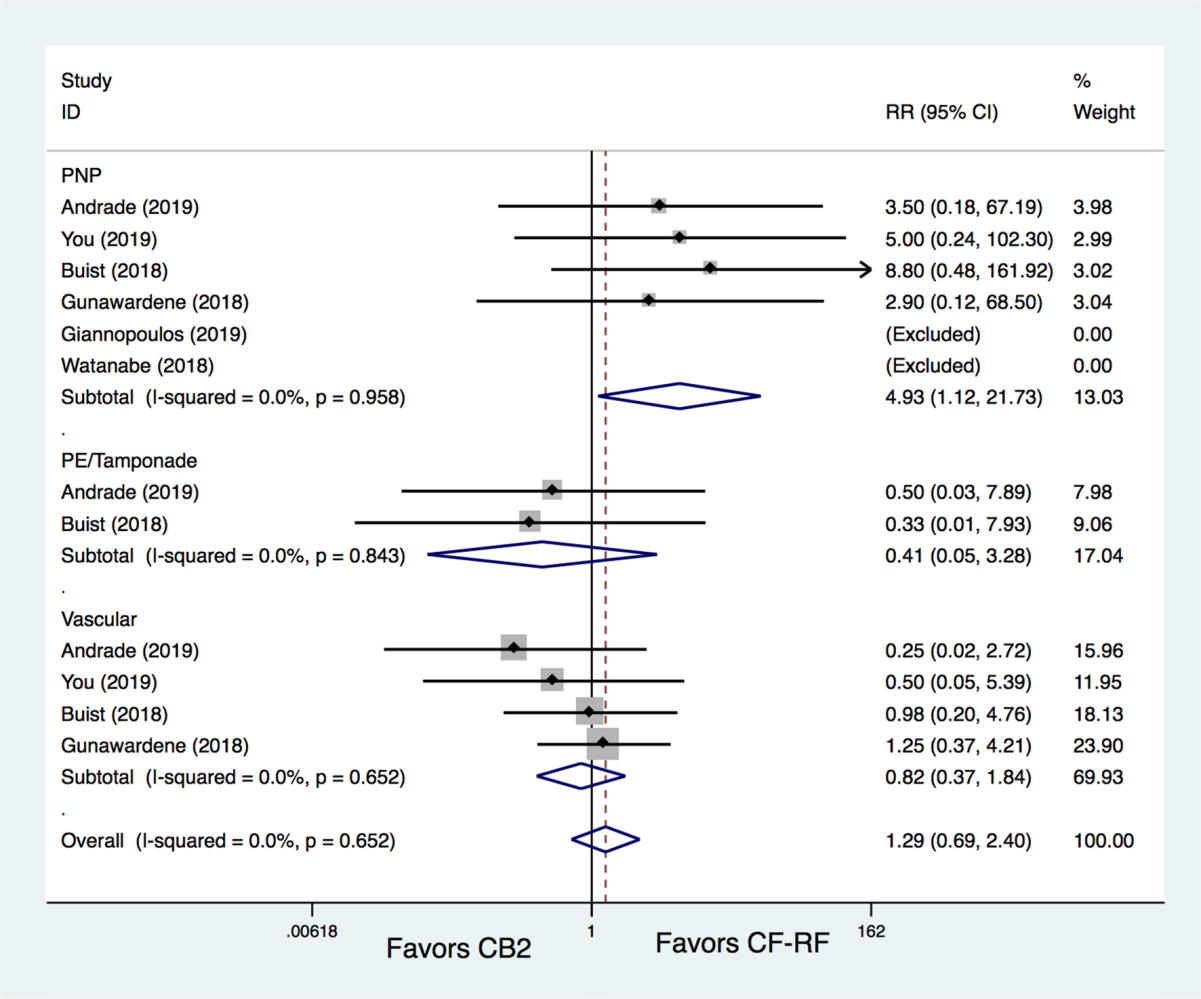
Subgroup meta-analysis for the outcome of complications. PN* p* = phrenic nerve palsy; PE = pericardial effusion; RR = relative risk.

Further subgroup analyses were conducted based on complication types, including PNP, pericardial effusion (PE)/tamponade and vascular complications. The results demonstrated that the CB2 group had significantly higher PNP rates than the CF-RF group (1.7% versus 0%, RR = 4.93, 95% CI 1.12–21.73, *p* = 0.035) (Fig. 5). All PNP events occurred in the CB2 group, and most of them were transient (70%). Additional subgroup analyses showed no difference between CB2 and CF-RF regarding either transient PNP (RR = 5.24, 95% CI 0.91–30.1, *p* = 0.063) or permanent PNP (RR = 3.50, 95% CI 0.18–67.19, *p* = 0.406). The occurrences of PE/tamponade and vascular complications were comparable between the CB2 and CF-RF groups (0.2% versus 0.5%, RR = 0.41, 95% CI 0.05–3.28, *p* = 0.398; 1.7% versus 2.7%, RR = 0.82, 95% CI 0.37–1.84, *p* = 0.632, respectively) (Fig. 5).

**Figure 6:**
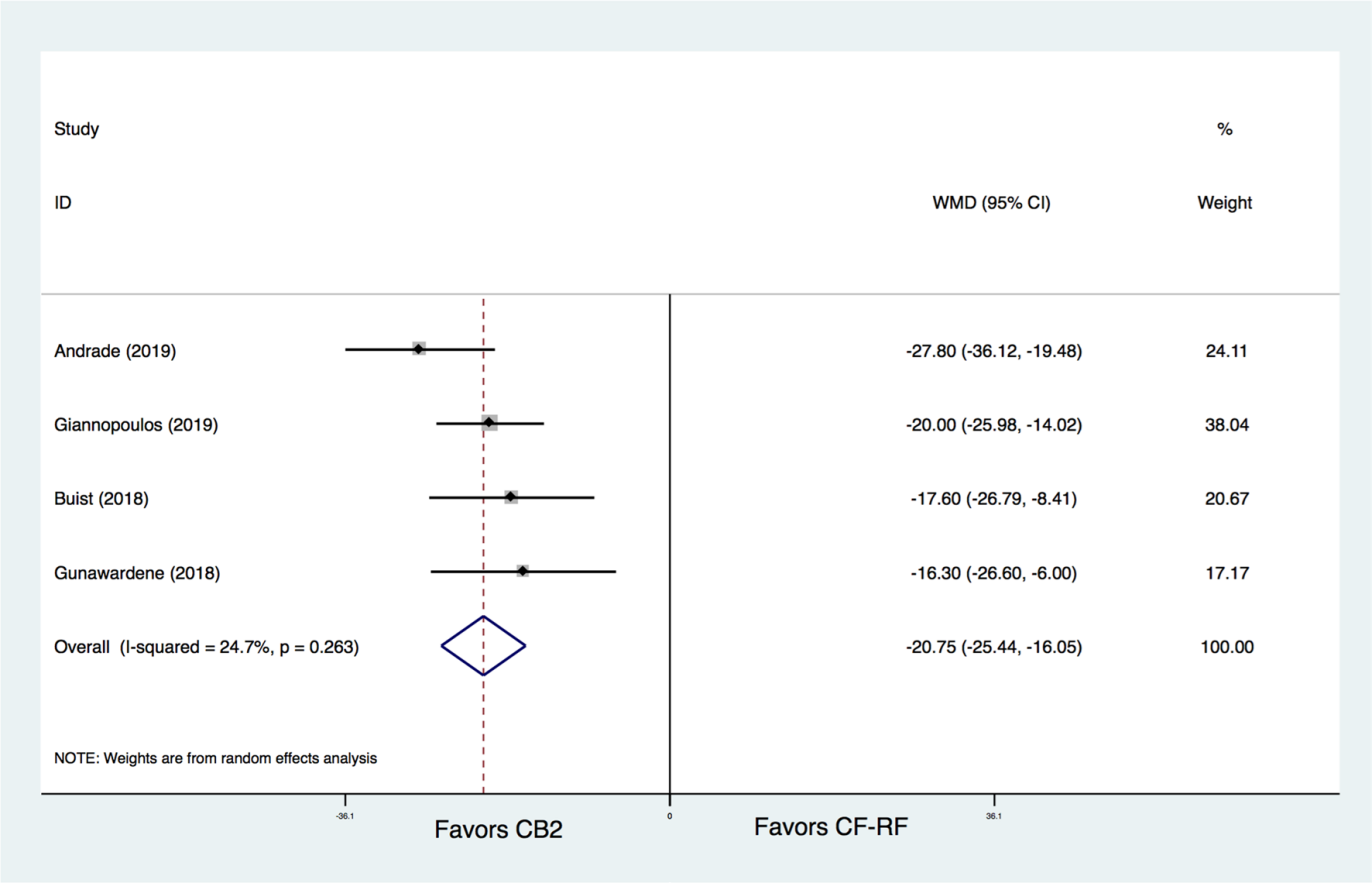
Meta-analysis for the outcome of procedure time. CF-RF = contact force radiofrequency ablation; CB2 = cryoballoon ablation (CBA) with a second-generation cryoballoon; WMD = weighted mean difference.

**Figure 7:**
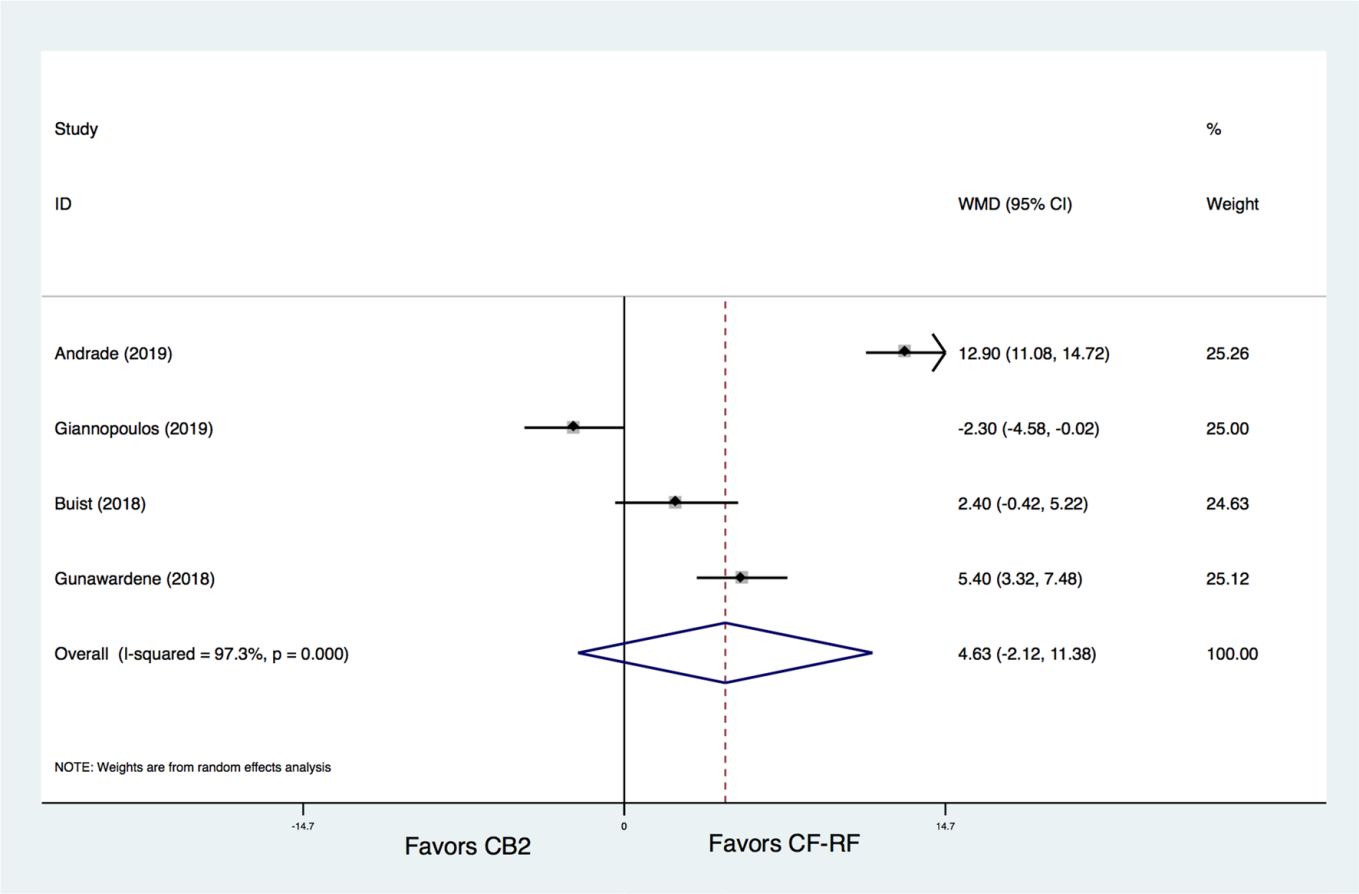
Meta-analysis for the outcome of fluoroscopy time. CF-RF = contact force radiofrequency ablation; CB2 = cryoballoon ablation (CBA) with a second-generation cryoballoon; WMD = weighted mean difference.

In addition, CB2 was found to have a significantly shorter procedure time than CF-RF (WMD = − 20.75 min, 95% CI − 25.44 ~ − 16.05 min, P < 0.001) (Fig. 6). In terms of fluoroscopy time, there was no significant difference between CB2 and CF-RF (WMD = 4.63 min, 95% CI − 2.12 ~ 11.38 min, *p* = 0.179) (Fig. 7). However, significant heterogeneity was detected (I^2^ = 97.3%), and further leave-one-out analysis was conducted. The results showed that when the study by Andrade et al^8^ was removed, the fluoroscopy time of CF-RF became significantly shorter than that of CB2 (WMD = 6.96 min, 95% CI 0.70–13.23 min, *p* = 0.029). However, the results were similar when the remaining trials were removed (WMD ranging from 1.84 min to 5.36 min).

## Discussion

### Main findings

The present study comprehensively evaluated the effectiveness, safety and procedural characteristics of CB2 and CF-RF using evidence only from RCTs. The results demonstrated that CB2 and CF-RF are comparable for the treatment of AF in terms of freedom from AT and total procedure-related complications. In addition, compared to CF-RF, CB2 treatment has a significantly shorter procedure time, but a higher risk of PNP.

### Effectiveness

PVI was introduced in 1988 by Haisaguerre et al. and has now become the cornerstone of AF^17^. RF has been a standard of care for PVI; however, it still has shortcomings, such as technical complexity, long procedure time, high rates of complications and long learning curve^18^. Newer catheter technologies and different energy sources, such as contact force technology for RF ablation and CBA, have been introduced in recent years. Among these, CBA has emerged as a valid alternative due to its simplicity, reproducibility, and similar effectiveness compared with RF^19^. In our previous work that comprehensively compared different catheter ablation interventions^20^, we found that CBA showed comparable clinical effectiveness and safety compared to RF ablation, which is also consistent with the largescale Fire and ICE trial^21^; however, the comparison between CB2 and CF-RF was not specifically focused on in this study^20^.

The study by Fortuni et al. found that CB can reduce the incidence of AF recurrence compared with RF ablation. However, in this study, mixed AF patient groups were included, and evidence from both observational studies and RCTs was analyzed^22^. In addition, most previous studies compared CBA with a first-generation cryoballoon (CB1) or CB2 with noncontact force radiofrequency (nCF-RF) ablation. CB2 and CF-RF were not further compared and evaluated, which may compromise the results^23^.

Structural improvements with CB2 can optimize the refrigerant distribution to make the freezing zone more uniform and address issues with abnormal PV anatomy^24–26^. However, it still has some inherent shortcomings, including ablation for non-PV triggers and complex fractionated atrial electrograms (CFAEs)^27^. In addition, the advent of contact-force technology for RF ablation could contribute to catheter contact and improve the success rate of PVI^28^.

Thus, the comparison between CB2 and CF-RF has more critical clinical significance. Previously, Jiang et al. compared CB2 with RF and found that CB2 treatment is associated with a significantly lower recurrence rate of AT; however, this study was based on evidence from observational studies, and a high heterogeneity among the trials was detected^10^. The present study has demonstrated that CB2 has comparable effectiveness compared with CF-RF, which is consistent with the previous meta-analysis by Ravi et al^11^. The study by Vogler also showed that endocardial PVI may have similar outcomes regardless of the techniques used^29^.

### Safety

The prevalence of major complications of CA for AF varies between 0.8% and 16.3% according to previously published studies^30–33^. Previous studies showed that, compared to RF ablation, CBA was associated with a high risk of procedure-related complications and a low risk of pericardial effusion or cardiac tamponade^34^. Other studies have also demonstrated that RF ablation has a relatively higher risk of pericardial effusion/cardiac tamponade than CBA^35^.

In this study, the major procedure-related complications were found to be comparable between CB2 and CF-RF (5.1% versus 4.4%). No significant difference was found in terms of procedure-related pericardial effusions/cardiac tamponade, which was consistent with previous studies^36^. Possible explanations may be that most of the previous studies included mixed comparisons between CB1 and RF ablation, and between CB2 and RF ablation, few of them included CF-RF groups. As contact force ablation has been proven to reduce the major complications compared to noncontact force ablation^37^, the risk ratio might be altered as the advanced contact force technology has been introduced and widely used in high-volume centers.

PNP has been reported as a common complication of CBA and almost appeared in the CBA groups both in our study and other published studies^38^. The high risk of PNP may be related to the inherent property of CBA, as well as the pulmonary vein and phrenic nerve anatomy. Advanced CB2 was reported to have an even higher incidence of PNP, which may occur due to its larger cooling area and higher efficiency^39^. However, it should be pointed out that PNP is usually transient, as demonstrated in this study, and can be largely prevented by the pacing of the phrenic nerve during the procedure^40^.

### Procedural characteristics

CB2 was found to have a significantly shorter procedure time than CF-RF, and this was consistent with the results of previous studies, which showed similar results in the comparison between CB1 and nCF-RF^41^. This is not surprising, as “one-shot” catheter ablation techniques, such as CBA, may require less manipulation time and fewer repeat ablations, which may make them better cost-effective approaches^42,43^. However, as a high-power short-duration (HPSD) approach has been utilized, the procedural duration should be greatly shortened.

Fluoroscopy times were found to be shorter in CBA than in RF ablation in previous studies^41,44^, which was not seen in our study or studies by other researchers^45^. Our study found no significant difference between CB2 and CF-RF in terms of fluoroscopy time. However, it should be noted that significant heterogeneity was detected for this outcome, and additional leave-one-out analysis even demonstrated a shorter fluoroscopy time in the CF-RF group than in the CB2 group. However, the heterogeneities were significant both for the overall analysis and the additional sensitivity analysis, which should be translated with caution. This is reasonable, as for patients with abnormal pulmonary veins, balloon placement may need prolonged time and a higher resolution, which greatly increases the exposure dose. However, the use of contact-force technology, combined with a three-dimensional electroanatomic mapping technique, could reduce the fluoroscopy time to a large extent. The introduction of intracardiac echocardiography even makes it possible for RF ablation procedures with zero fluoroscopy exposure, which seems impossible for “one shot” techniques such as CBA^46^.

Many new approaches and techniques have also been increasingly introduced in recent years to increase the effectiveness and safety of PVI for AF patients. Gupta D et al. has compared different catheter ablation devices, including CF-RF with ablation index, CF-RF, nCF-RF, CB1 and CB2, and found that CF-RF with an ablation index is associated with the highest rate of freedom from AT compared to other approaches^47^. The combination of CB2 and RF ablation was also found to be safer and more effective for treating PerAF^24^.

For the improved and most widely used techniques of CF-RF and CB2, further large-scale studies are still warranted to comprehensively evaluate their clinical values and compare them with other newly introduced methods to provide an up-to-date optimal recommendation. For example, CB2 might be better suited for patients with impaired heart function, while CF-RF might be better suited for patients with specific anatomic variations of the PV.

### Study limitations

The present meta-analysis was performed based on six high-quality RCTs with 987 patients; however, there are several limitations. First, the number of trials included and the sample size were relatively small, as we only included evidence from RCTs. However, we intended from the start to use only high-quality evidence only for meta-analysis to make a convincing conclusion. Second, this was a study-level meta-analysis, which had important limitations compared to an individual patient-level data meta-analysis and may lead to biased assessments, as patient characteristics may be related to treatment effects. The protocol of this meta-analysis has yet to be registered in an international registry. Third, mixed AF populations (94% PAF vs. 6% PerAF) were included in the analysis. Although subgroup analyses demonstrated similar results for PAF and PerAF patient groups, considerable heterogeneity was detected for the analysis of PerAF; thus, the interpretation should be taken with caution. In addition, endpoint definitions and AT recurrence monitoring protocols across trials were nonuniform, which may cause possible bias. Finally, the follow-up durations were abbreviated, as the longest was 12 months, which may be insufficient to evaluate late AF recurrence and the long-term success rate. Thus, large-scale RCTs are still warranted to confirm the findings of this study.

## Conclusions

For AF patients, CB2 and CF-RF treatment have comparable effectiveness and safety in terms of freedom from AT and procedure-related complications. CB2 treatment is associated with a shorter procedure time than CF-RF, but it is also associated with a higher risk of PNP. Further studies are needed to compare these two techniques in real-world clinics, to provide an up-to-date recommendation.

## Ethics declarations

Not applicable as this is a meta-analysis of previously published papers.

## Data Availability

Available upon request to the corresponding author Xinbin Zhou (zhouxinbin@zcmu.edu.cn).
